# Clinical and genomic evaluations of a persistent fatal SARS-CoV-2 infection in a goods syndrome patient: a case report

**DOI:** 10.1186/s12879-024-09105-6

**Published:** 2024-02-19

**Authors:** Payam Tabarsi, Ali Maleki, Zahra Abtahian, Alieh khabbaz, Zahra Fereydouni, Jahangir Rezaie, Mahsa Tavakoli, Parastoo Yektay Sanati, Mostafa Salehi-Vaziri

**Affiliations:** 1 Clinical Tuberculosis and Epidemiology Research Center, National Research Institute of Tuberculosis and Lung Diseases (NRITLD), Shahid Beheshti University of Medical Sciences, Tehran, Iran; 2 COVID-19 National Reference Laboratory (CNRL), Pasteur Institute of Iran, Tehran, Iran; 3 Department of Influenza and other Respiratory Viruses, Pasteur Institute of Iran, Tehran, Iran; 4 Department of Arboviruses and Viral Hemorrhagic Fevers (National Reference Laboratory), Pasteur Institute of Iran, 69 Pasteur Ave, 1316943551 Tehran, Iran

**Keywords:** Good syndrome, SARS-CoV-2, COVID-19, Autoimmune diseases

## Abstract

The coronavirus disease of 2019 (COVID-19) resulted from an infection by severe acute respiratory syndrome coronavirus 2 (SARS‑CoV‑2) which is the main cause of acute respiratory distress syndrome (ARDS) in global population from 2019 on. It may contribute to higher rate of death among the patients with immunodeficiency based on recent reports. In addition, Good syndrome (GS) as a result of thymoma removal might cause in some long-lasting microbial infections. We described clinical aspects and viral mutations on a case of GS suffering from COVID-19. A 46-year-old man with fever, common respiratory disease symptoms and positive COVID-19 polymerase chain reaction (PCR) test, with the history of thymoma removal surgery was admitted to Masih Daneshvari Hospital, Tehran, Iran. Lung radiographs and oxygen saturation measurement disclosed considerable implication resulted in application of several anti-microbial medication. The delta variant (B.1.617.2 (21 J Clade)) was the strain isolated from the patient by sequencing methods done by the COVID-19 National Reference Laboratory (CNRL), Pasteur Institute of Iran, while the dominant strain circulated mostly among population was Omicron (B.1.1.529) at the time of sampling. Unfortunately, the patient had passed away a month later by sudden respiratory failure progressed in refractory septic shock. Despite the fact that opportunistic infections may lead the GS patients to a major health problematic condition, unusual persistent of infections such as non-dominant variant of SARS-Cov-2 could be observed through the disease timeline. Therefore, a fully screening of thymoma plus intra-host evolution monitoring of SARS-CoV-2 is highly recommended in immunocompromised patients.

## Introduction

From late 2019 and early 2022 on, the world has been struggling with the ADRS called coronavirus COVID-19 which is caused by SARS-CoV-2 [1]. Over 762 million confirmed cases of COVID-19 and about 6.8 million related deaths were reported globally by April 13, 2023 [2]. In comparison with the general population, COVID-19 is associated with higher morbidity and mortality in immunodeficient patients such as cancer and AIDS [3, 4]. Good syndrome (GS) is an uncommon autoimmune disease which is characterized by thymoma associated with combined B and T cell immunodeficiency, hypogammaglobulinemia and therefore the elevated risk of bacterial, fungal and viral infections [5]. Persistent or relapsing SARS-CoV-2 infection has been recorded in immunocompromised individuals [6, 7]. Recently, relapsing COVID-19 has been reported as a manifestation of GS [8]. Despite a few case reports of concurrent COVID-19 and GS, there is little data about the clinical features of COVID-19 and mutation analysis SARS-CoV-2 of in patients with GS. Here, we present a persistent SARS-CoV-2 infection in an Iranian patient with GS and describe clinical characteristics. In this study, we highlighted that the immunocompromising condition could alter the revolution of SARS-CoV-2 and a situation like long-COVID may arise. In such a way that a patient had a nearly stable or a little increasing viral load for more than one month.

## Case presentation

**Fig. 1:**
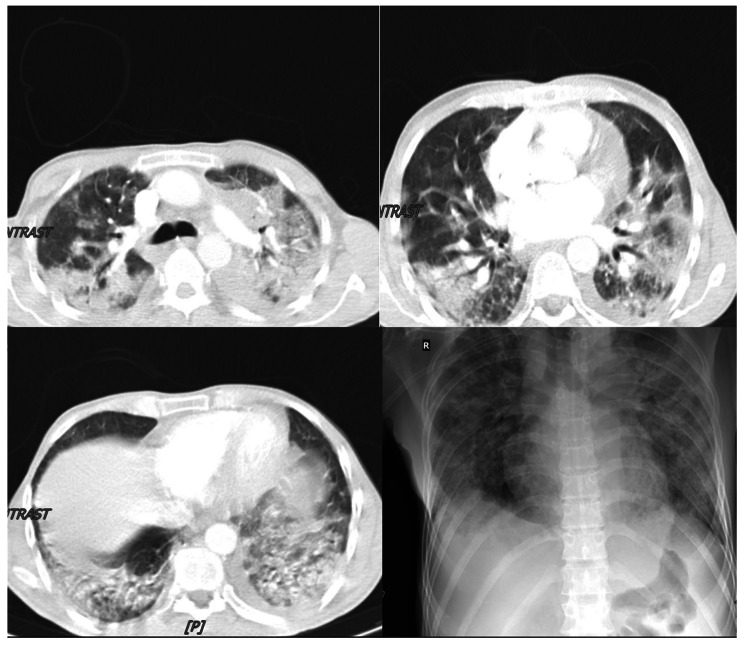
Chest CT scan of the patient on 06/10/2022

**Fig. 2:**
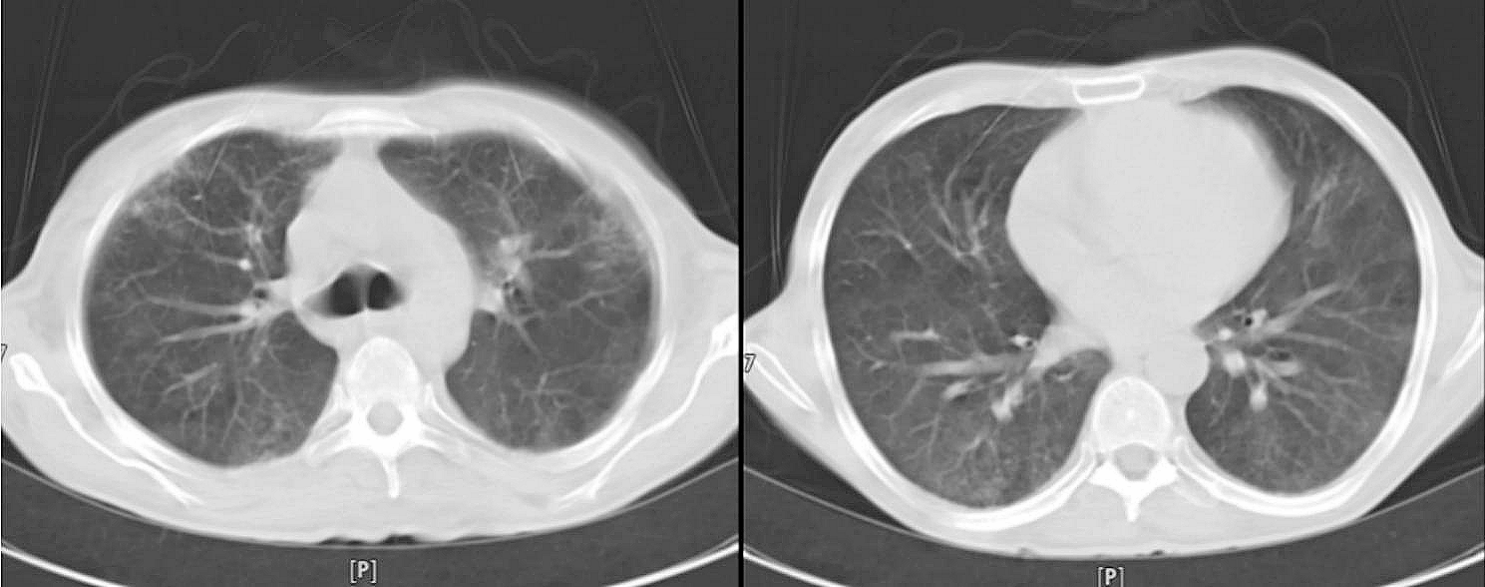
Spiral chest CT scan of the case 1, 06/28/2022. The Fig. 2 concludes his test outcome generally. Radiographic abnormality improved dramatically

On July 6, 2022, a 46-year-old man was admitted to Masih Daneshvari Hospital, Tehran, Iran due to, fever, cough, respiratory distress, and a positive COVID-19 Real-Time PCR (Table 1). Despite receiving 3 doses of COVID-19 Sinopharm vaccine (Sinopharm BIBP COVID-19 vaccine) on December 21, 2021. For confirmation of SARS-CoV-2 infection, COVID-19 Real-Time PCR test was performed which became positive. Chest radiography revealed an extensive involvement of both lungs (Fig. 1) and his oxygen saturation was 65%. Broad spectrum antibiotics, anti-fungal, remdesivir and dexamethasone was started and one dose of Tocilizumab was administered, as well. He had experienced a removal operation of thymoma on July 11th,2021 which led to the suspicion of GS. Therefore, a comprehensive immunologic investigation via ELISA and flow cytometry was performed and due to hypogammaglobulinemia and significant decreased number of CD19 and CD4, diagnosis of good’s syndrome was established. Table 1 represents the results of laboratory investigations for GS. IVIG 40 gr was administered and patient gradually improved (Fig. 2). The SARS-CoV-2 infection was observed for approximately one month due to his immunocompromised condition. In this regard nasopharyngeal swab samples were collected weekly (from 06.14.2022 to 07.19.2022) and sent to the COVID-19 National Reference Laboratory (CNRL) at Pasteur Institute of Iran. RNA extraction was performed by RNJia Virus Kit (ROJE, Iran) and COVID-19 Real Time PCR test was performed using Novel Coronavirus (2019-nCoV) Nucleic Acid Diagnosis Kit (Sansure Biotech, China) according to the manufacturer’s instructions [9]. Table 2 shows that during the final week of Ct value monitoring, not only did the Ct value increase, but it also demonstrated a 3-unit reduction 3 (the SARS-CoV-2 N gene CT value), indicating a roughly 10-fold increase in viral load. This is because the Ct value and viral load are inversely correlated; for every 3.3 increases in Ct value, there is a corresponding 10-fold decrease in starting RNA molecules [10].

**Table 1:** The clinical and immunological marker’s profile of the case

Row	Factor	Result	Unit
1	WBC	3200	cells/µL
2	HG	10.5	g/dL
3	PLT	270,000	cells/µL
4	Urea	24	mg/dL
5	Cr	0.7	mg/dL
6	LDH	813	units/L
7	CRP	64	mg/L
8	B/C	Negative	-
9	U/C	Negative	-
10	Ferritin	1400	ng/mL
11	D-dimer	950	mg/L
12	PCR COVID-19	Positive	-
13	Smear of sputum for AFB	Negative	-
14	Culture of Sputum for Bacteria	Negative	-
15	Culture of Sputum for Fungi	Negative	-
16	IgG	445	mg/dl
17	IgM	22	mg/dl
18	IgE	1	mg/dl
19	IgA	17	mg/dl
20	CD_19_	1	%
21	CD_20_	1	%
22	CD_4_	22	%
23	CD_8_	42	%

Abbreviations: WBC, white blood cell; HG, Hemoglobin; PLT, platelet; Cr, creatinine; LDH, lactate dehydrogenase; CRP, c-reactive protein; B/C, blood culture; U/C, urine culture; AFB, acid-fast bacilli; IgG, Immunoglobulin G; IgM, Immunoglobulin M; IgE, Immunoglobulin E; IgA, Immunoglobulin A; CD, cluster of differentiation

**Table 2:** Monitoring of SARS-CoV-2 infection by Real-Time PCR test

Row	Date of Sampling	N gene Ct	ORF 1 gene Ct	Patient Status
1	06/14/2022	25	28	Hospitalized
2	06/21/2022	18	19	Hospitalized
3	06/28/2022	18	19	Hospitalized
4	07/06/2022	19	19	Hospitalized
5	07/13/2022	19	19	Hospitalized
6	07/19/2022	16	17	Hospitalized

The observation of persistent SARS-CoV-2 infection in the patient persuaded us to investigate the genetic mutations of the virus. For the evaluation of viral genomic profile, five nasopharyngeal samples were sequenced via Oxford Nanopore Technology (ONT, UK) Next Generation Sequencing (NGS) machine with Midnight RT-PCR Expansion (EXP-MRT001) and Rapid Barcoding Sequencing Kit (SQK-RBK110.96). Variant assignment and mutation detection were done by the Nextclade online tools (https://clades.nextstrain.org/). The sequence was submitted to the GISAID with the accession numbers of EPI_ISL_17585020/1/2/3 and EPI_ISL_17585036. The sequencing result was far more surprising than the SARS-CoV-2 persistence. The patient was infected with a strain belonged to the delta variant (B.1.617.2 (21 J Clade)), while the variant data was circulation in Iran year before Feb 2022 and at the time of sampling from the patient, the dominant variant in the country was Omicron (B.1.1.529).

For detection of simulations infections with other respiratory viruses a commercial multiplex Real Time PCR assay targeting 17 viruses including SARS-CoV-2, Flu/A, Flu/B, Flu H1N1, HCoV-NL63, HCoV-229E, HCoV-HKU1, HCoV-229E, HCoV-OC43, PIV1/2/3, AdV, hRV, HBoV1/2/3, hMPV, and RSV (HiTeq 17 Viro Respiratory pathogens One-step RT-PCR kit (GeneovA, Iran)) which tested negative for all viruses, apart from SARS-CoV-2.

Unfortunately, one month later patient developed respiratory failure and was intubated. The patient still remained in the hospital. In less than a day, he suddenly experienced a respiratory distress and reduction in oxygen saturation. Since the patient’s condition was unstable, a bronchoscopy was not possible. It is thought that a COVID-19-related illness resulted in refractory septic shock, and the patient passed away. However, hospital acquired infections cannot be completely ruled out.

## Discussion

Goods syndrome (GS) is a rare adult-onset combined immunodeficiency that occurs in patients with thymoma [4]. Most cases happen in the 40–70 age range. Despite the GS was initially discovered in 1957, its pathogenesis remains unclear. Only 6 to 10% of patients with thymoma develop GS [11]. The two most common immunologic abnormalities are decreased or nonexistent B cells and hypogammaglobulinemia. Other abnormalities include decreased T-lymphocytes, inversion of the CD4+/CD8 + ratio, and functional defects in cell-mediated immunity [12].

The immunoglobulin levels as well as B and T cell subsets should be assessed in all thymoma patients. Repeat immunoglobulin measurements should be taken on an annual basis if these are normal because cases of progressive immunodeficiency have been reported [5]. Infections remain still the major cause of death in patients with GS. The predominant pathogen responsible for GS patients’ deaths is encapsulated bacteria, along with opportunistic viruses and fungi [4, 13]. Very few cases of GS with COVID-19 have been reported since the onset of COVID-19. The majority had severe courses [8, 14–17]. At least in two cases, a relapsing nature of disease has been reported. A recent study found that a patient initially tested positive for SARS-CoV-2, then tested negative within a week, and then tested positive again. Notably, the patient was infected with a SARS-CoV-2 variant that was prevalent five months ago and different from the circulating variant in the time of sampling [8]. Death rates continue to be high even with antimicrobial therapy and immunoglobulin replacement [14, 15, 17].

The presented case has some unique features. First, during one year all PCR tests were positive for SARS-CoV-2. At admission to the Masih Daneshvari hospital, the SARS-CoV-2 variant was delta which was dominant variant in nearly one year ago, while the Omicron was predominant variant at the time of admission. Second, goods syndrome was not diagnosed till admission. It shows that all thymoma patients must be screened for immunodeficiency, especially when present with unusual infections.

Severe, fatal and recurrent case reports of SARS-CoV-2 infections in patient suffering from GS have been reported during the COVID-19 pandemic [8, 14–18]. In our case an unusual persistent infection of SARS-CoV-2 was observed. Detection of a strain belonging to delta variant which was dominant in Iran approximately one year before sample collection from our case suggests that he was infected with delta variant at least before Feb 2022 and his immune system was unable to clear the virus during this period of time. In a recent case report from Singapore, SARS-CoV-2 persistence was identified as identical SARS-CoV-2 strains were detected in respiratory specimens collected over the course of disease [8].

GS patients faced a range of outcomes following COVID-19 infection. While some individuals experienced a favorable recovery [14], others developed severe complications that resulted in fatalities. Building upon the existing knowledge of COVID-19 infection in GS patients [17, 18], we present a compelling case highlighting the prolonged persistence of SARS-CoV-2 in a GS patient, extending from several months to even a year.

Persistent SARS-CoV-2 infections in immunocompromised patients have been implicated in the accumulation of mutations and consequently may result in the emergence of new variants [19]. Nevertheless, our case’s mutation profile did not result in the emergence of a new variant, similar to the findings of the Wee et al. study [7]. In fact, the following samples of the case had additional nucleotide substitutions, compared to the first sample. This showed the intra-host evolution should be monitored in the immunocompromised patient with persistent SARS-CoV-2 infection. Prolonged SARS-CoV-2 infections in patients with a history of thymoma should provoke one to investigate for GS, accelerating early diagnosis and timely treatment.

## Data Availability

The datasets used and/or analyzed during the current study available from the corresponding author on reasonable request.
